# An unusual cause of large bowel obstruction: are we aware of this?

**DOI:** 10.11604/pamj.2020.36.223.24718

**Published:** 2020-07-28

**Authors:** Maria Sotiropoulou, Nikolina Stavrinou, Michail Vailas, Paraskevi Alexakou, Michail Psarologos, Panagiotis Metaxas, Michael Economou, Christine Vourlakou, Stylianos Kapiris

**Affiliations:** 1 Third Department of Surgery, Evangelismos General Hospital, Athens, Greece,; 2 Department of Pathology, Evangelismos General Hospital, Athens, Greece,; 3 First Department of Surgery, National and Kapodistrian University of Athens, Laikon General Hospital, Athens, Greece,; 4 Department of Gastroenterology, Euroclinic, Athens, Greece

**Keywords:** Burkitt lymphoma, lymphoma, obstruction, surgery

## Abstract

Primary lymphomas of the colon account for 0.5% of all primary colon malignancies. Burkitt´s lymphoma is a B-cell lymphoma with aggressive clinical behavior. Herein, we describe a case of a male patient who presented with signs of large bowel obstruction, underwent surgery and found to suffer from Burkitt´s lymphoma of the ileocecal region. The histopathological examination was indicative for Burkitt´s lymphoma. To the best of our insight this is one of the few reported cases of such type of lymphoma in an adult patient presenting with bowel obstruction. Burkitt´s lymphoma is a rare malignancy in adults affecting gastrointestinal tract. It has a high proliferation potential and can rapidly progress to advanced disease. Early diagnosis is necessary to prevent complications and improve overall prognosis.

## Introduction

Primary lymphomas of the colon account for 10-20% of all gastrointestinal lymphomas and approximately 0.5% of primary colon malignancies. Most gastrointestinal lymphomas are of B-cell origin that also applies to Burkitt´s lymphoma (BL), an aggressive type of malignancy which usually is diagnosed in children and immunocompromised patients, with few cases reported in middle-aged or elderly people [1]. Three distinct variants of BL have been recognized; endemic, sporadic and immunodeficient. Endemic usually appears in African countries usually in children, whereas sporadic form shows no predilection as far as geographical areas are concerned [2]. On the other hand, immunodeficient variant has been reported in patients suffering from human immunodeficiency virus (HIV), in solid organ recipients as well as in patients with congenital immunodeficiency diseases. Presentation in elderly and adult population is quite rare with few cases reported in the medical literature [3]. Herein, we describe a case of a 63-year old patient who presented in the emergency department with signs of large bowel obstruction, underwent curative surgery and found to suffer from BL of the ileocecal region. To the best of our insight this is one of the few reported cases of such type of lymphoma in an adult patient presenting with bowel obstruction.

## Patient and observation

**Figure 1:**
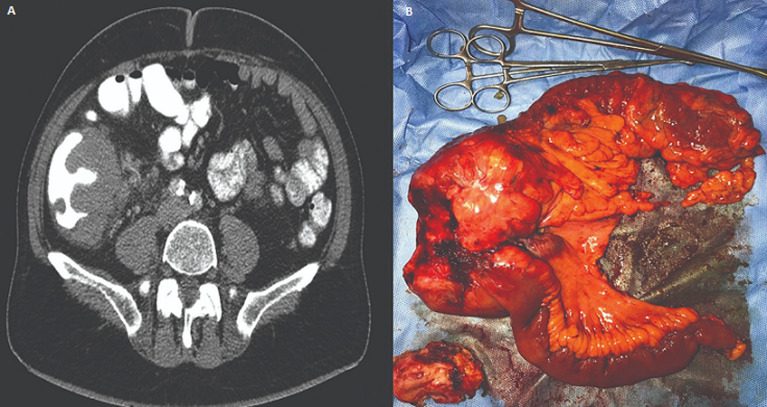
A) computed tomography scan showing a large mass constricting the bowel lumen at the ileocecal junction; B) photo of the resected surgical specimen (right hemicolectomy)

A previously healthy 63-year-old Caucasian male patient, with no significant past medical history, presented to the emergency department suffering from abdominal distension, nausea and vomiting. The aforementioned symptoms started abruptly approximately six hours before his admission to the hospital. He denied taking any prescription or over-the-counter medications. Two years prior he had undergone lower gastrointestinal endoscopy, which showed no mucosal abnormalities. Clinical examination revealed a well-nourished male patient with no previous abdominal surgical scars. The patient was diaphoretic and his vital signs were as follows: blood pressure 100/75mmHg, 115bpm, SaO2 95%, temperature 37.4°C. Blood counts were within normal limits, except for creatinine levels reaching 1.7mg/dl. The rest of biochemical profile showed nothing remarkable. Significant abdominal dissension was noted along with hyperactive bowel sounds on auscultation. X-rays of the chest and abdomen were indicative for large bowel obstruction, a finding that was confirmed by computed tomography (CT) scan (Figure 1), which also revealed a large mass of the ascending colon constricting the bowel lumen, extending in a length of about 14cm with minimal involvement of the terminal ileum.

**Figure 2:**
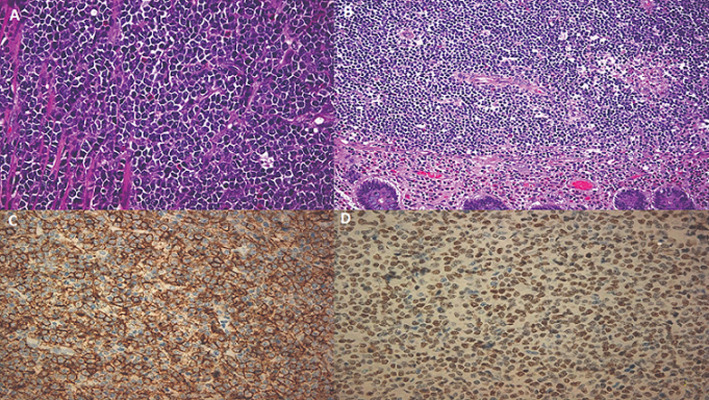
A) monomorphic neoplastic lymphoid cells and interspersed histiocytes consistent with the pathognomonic “starry sky” appearance of BL; B) infiltration of submucosa and complete destruction of the normal colonic architecture from BL (H-E x 100); C) immunohistochemistry showing CD20 stain positivity (×100); D) C-myc positivity in >80% of neoplastic cells

The patient was taken to the operating room after written consent was obtained. He underwent open right hemicolectomy with en-mass resection of a small part of abdominal wall with primary anastomosis of terminal ileum and transverse colon. His postoperative course was uneventful and he was discharged five days after the surgical operation. Histopathological examination was indicative for extensive malignant infiltration of the colon and terminal ileum from Burkitt’s lymphoma with small atypical lymphocytes with round to irregular nuclei, prominent nucleoli and scant cytoplasm consistent with the pathognomonic “starry sky” appearance of BL. Immunochemistry reported positive results for CD10, CD20, Pax-5, bcl-6 and negative for markers like CD5, CD23, cyclin-D1 and CD30. The cytogenetical analysis revealed proto-oncogene (c-myc) rearrangement (>80%), BCL-2 (-), Ki-67 100% (Figure 2). The specimen showed negativity for EBV. The patient underwent bone marrow biopsy and full-body CT scan for a further evaluation of the disease. Bone marrow biopsy demonstrated normal proliferation and CT scan did show possible localization of the disease in the left adrenal along with underdetermined findings in the head of the pancreas and spleen. Multidisciplinary team suggested patient should follow CVAD combined chemotherapy (cyclophosphamide, doxorubicin, vincristine and prednisone). At 6-month follow-up, the patient remains alive and free of suspicious for malignancy lesions.

## Discussion

Burkitt´s lymphoma is a highly aggressive malignancy usually affecting children and young adults. In more elderly population its incidence is low, with patients 20-30 years of age showing a figure of 0.6 per million [4]. The first reported case of BL was observed in young children from Africa by Burkitt in 1958. This disease shows predilection for white males in about 71-89% of cases with three distinct variants, endemic, non-endemic and immunodeficient form usually in HIV patients [5]. BL of the gastrointestinal tract most commonly arises in the ileocecal region, where an increased concentration of lymphoid tissue is present. Clinical presentation includes abdominal pain and distention such as in our case, constipation, bowel obstruction, back pain, ascites and melena. There are no characterizing imaging findings and quite often colon BL is diagnosed after emergency surgical operation and histopathologic examination of the resected specimen [6]. Burkitt´s lymphoma involvement most commonly is extranodal in children and adolescents (81% extranodal) with prevalence that reaches 0.6% of all pediatric tumors, whereas in adults nodal disease occurs more frequently (89% nodal). While endemic form of BL involves mostly the facial bones, particularly the jaw and maxilla, especially in young population and is related with Epstein-Barr virus (EBV) infection, in non-endemic form which is reported mainly in western countries often appears as a large abdominal mass [7].

BL is notable for remarkable uniformity of nuclear size and contour along with chromosomal (8: 14) translocation and overexpression of c-myc, adopting a pattern of characteristic 'starry-sky' pattern with sheets of monomorphic neoplastic lymphoid cells and interspersed histiocytes. Burkitt´s lymphoma typically expresses pan-B-cell antigens like CD19, CD20, CD22, and CD79a and co-expresses CD10, Bcl-6, CD43 and p53. On the other hand, BL shows negativity for CD5, CD23, Bcl-2, CD138, or TdT. The proliferation fraction is nearly 100% [8]. Burkitt´s lymphoma has an extremely aggressive behavior with doubling time that ranges between 24-48 hours. As a consequence of this, prompt diagnosis and initiation of therapy in order to prevent the dissemination of disease is imperative [8]. Treatment usually consists of high dose chemotherapy regimens such as cyclophosphamide, doxorubicin, vindesine, bleomycin and prednisone (ACVBP) or cyclophosphamide, doxorubicin, vincristine and prednisone (CHOP) with high rates of response [9]. Surgical treatment is reserved for complicated abdominal cases like in our case, despite the fact that the utility of aggressive surgical management in children with BL, even in the setting of abdominal emergencies, has been questioned in several studies. Five-year survival rates of children with localized disease are approximately 90%, reaching 80-84% in those with advanced forms of disease. Cure rates for adults treated with chemotherapy range between 65-80% [10].

## Conclusion

In conclusion, BL is a rare malignancy in adults affecting gastrointestinal tract, mainly in ileocecal region. Thanks to the better understanding of the biology of BL, there has been significant improvement in chemotherapeutic drugs. In the era of modern chemotherapy, BL may readily respond to treatment, provided that it is diagnosed before extensive dissemination of disease occurs. Therefore, a high clinical suspicion along with prompt diagnosis and treatment based on multidisciplinary approach is required. Surgical therapy may play a role in the management of advanced complicated cases such as bowel obstruction or perforation, but chemotherapy remains the mainstay of treatment.
